# Comparative Metagenomics of Palearctic and Neotropical Avian Cloacal Viromes Reveal Geographic Bias in Virus Discovery

**DOI:** 10.3390/microorganisms8121869

**Published:** 2020-11-26

**Authors:** Daniel A. Truchado, Alejandro Llanos-Garrido, David A. Oropesa-Olmedo, Belén Cerrada, Pablo Cea, Michaël A. J. Moens, Esperanza Gomez-Lucia, Ana Doménech, Borja Milá, Javier Pérez-Tris, Daniel Cadar, Laura Benítez

**Affiliations:** 1 Department of Genetics, Physiology and Microbiology, Faculty of Biology, Complutense University of Madrid, José Antonio Novais, 12, 28040 Madrid, Spain; doropesa@ucm.es (D.A.O.-O.); bcerrada@ucm.es (B.C.); pcea@ucm.es (P.C.); lbenitez@ucm.es (L.B.); 2 Department of Biodiversity, Ecology and Evolution, Faculty of Biology, Complutense University of Madrid, José Antonio Novais, 12, 28040 Madrid, Spain; jperez@ucm.es; 3 Department of Organismic and Evolutionary Biology, Faculty of Arts & Sciences, Harvard University, 38 Oxford St, Cambridge, MA 02138, USA; allanosgarrido@fas.harvard.edu; 4 Fundación de Conservación Jocotoco, Lizardo García E9-104 y Andrés Xaura, Quito 170143, Ecuador; m.moens@bio.ucm.es; 5 Department of Animal Health, Faculty of Veterinary Medicine, Complutense University of Madrid, Avda. Puerta de Hierro s/n, 28040 Madrid, Spain; duato@ucm.es (E.G.-L.); domenech@ucm.es (A.D.); 6 National Museum of Natural Sciences, Spanish National Research Council (CSIC), 28006 Madrid, Spain; b.mila@csic.es; 7 Bernhard Nocht Institute for Tropical Medicine, WHO Collaborating Centre for Arbovirus and Haemorrhagic Fever Reference and Research, National Reference Centre for Tropical Infectious Diseases, 20359 Hamburg, Germany; danielcadar@gmail.com

**Keywords:** wildlife virome, avian virome, Neotropical birds, animal viruses, remote areas, biodiscovery

## Abstract

Our understanding about viruses carried by wild animals is still scarce. The viral diversity of wildlife may be best described with discovery-driven approaches to the study of viral diversity that broaden research efforts towards non-canonical hosts and remote geographic regions. Birds have been key organisms in the transmission of viruses causing important diseases, and wild birds are threatened by viral spillovers associated with human activities. However, our knowledge of the avian virome may be biased towards poultry and highly pathogenic diseases. We describe and compare the fecal virome of two passerine-dominated bird assemblages sampled in a remote Neotropical rainforest in French Guiana (Nouragues Natural Reserve) and a Mediterranean forest in central Spain (La Herrería). We used metagenomic data to quantify the degree of functional and genetic novelty of viruses recovered by examining if the similarity of the contigs we obtained to reference sequences differed between both locations. In general, contigs from Nouragues were significantly less similar to viruses in databases than contigs from La Herrería using Blastn but not for Blastx, suggesting that pristine regions harbor a yet unknown viral diversity with genetically more singular viruses than more studied areas. Additionally, we describe putative novel viruses of the families *Picornaviridae*, *Reoviridae* and *Hepeviridae*. These results highlight the importance of wild animals and remote regions as sources of novel viruses that substantially broaden the current knowledge of the global diversity of viruses.

## 1. Introduction

The current knowledge about the virome of wild animals is still incipient. During the last 30 years, zoonotic viruses with an origin in wildlife have been the main cause of disease outbreaks, sometimes pandemic [1]. This rapid, global spread of new viruses has revealed our vulnerability to emerging diseases that have caused a great number of negative effects both in human health and the economy [2]. In order to prevent future outbreaks, the search for novel viruses with a zoonotic potential in wildlife has become one of the main objectives of the One Health initiative [1,3]. Furthermore, understanding the virome of wild animals is not only important to detect potential novel zoonotic viruses before their emergence. In the last years, several studies analyzing viral diversity have challenged the view of viruses as only pathogens and have demonstrated that they constitute symbiotic microorganisms in the majority of the cases, even producing positive effects in their hosts [4]. Therefore, research on viral diversity of wildlife is necessary to have a complete view of the global virome, while it will help understand virus–host relationships and virus ecology. However, if we want to characterize the viral diversity of wildlife accurately, discovery-driven approaches are the optimal way of doing so. Describing novel viral strains while studying traditional hosts is no longer sufficient, and specific designs that allow a greater efficiency of virus discovery are needed. Additionally, expanding the scope of virus discovery to non-canonical hosts and remote regions is paramount to significantly increase the current knowledge of viral diversity. 

With around 10,000 described species (which may double according to phylogenetic diversity [5]), the Class Aves is the most diverse tetrapod clade, inhabiting every continent across the globe. This ubiquitous presence increases their exposure to diverse microorganisms [6] and, together with their diverse ecologies, make birds good candidates for microorganism circulation in different ecosystems. For example, migratory birds connect ecosystems that are separated by hundreds of kilometers, carrying parasites from their breeding areas to their wintering sites and vice versa [7,8]. Moreover, they may spread large quantities of a great diversity of viruses during a long period of time [9] without any clinical signs [9,10], and their tendency to roost and feed in heterospecific groups favors the occurrence of inter-species pathogen transmission and the emergence of novel viruses [11]. However, little is known about the virome of wild birds compared to their microbiome [12].

Despite the importance of increasing the knowledge about avian viruses, discovery-driven approaches to characterizing their global diversity have been rare. The majority of studies of wild bird viruses have focused on the surveillance of specific viruses that produce zoonotic infections or on those that cause massive mortalities such as highly pathogenic avian influenza virus, Newcastle disease virus, West Nile virus or Usutu virus [10,13,14,15]. Only a few studies analyze the complete virome of wild bird populations, being mainly aimed at waterfowl [10,16,17,18]. At the same time, the virome of wild passerine populations has never been analyzed, even though they constitute approximately 60% of avian diversity [19]. The only report of a wild passerine’s virome to date comes from the cloacal sample of one individual of the species *Sicalis flaveola* [20]. Thus, studying the virome of understudied wild bird populations will provide us with novel information about animal viruses that may be useful in the future by better preparing us for possible viral outbreaks or spillovers.

Apart from viruses that may cause disease, analyzing the virome of wild birds living in remote regions is especially interesting as it could provide useful knowledge about new virus–host relationships or the ecology of viruses circulating in ecosystems rarely disturbed by humans. The Guianan shield is one of such remote areas. Located in the Neotropics, this region is one of the main hotspots of avian diversity in the world, with more than 700 documented species [21]. The Guianan shield, and particularly French Guiana, is sparsely inhabited by humans and, therefore, anthropic impact is scarce. On the other hand, human impact in French Guiana, albeit rather low, can be sufficient to introduce novel viruses that might put its unique avifauna at risk. Anthropic impact on world avian population has involved a dramatic increase in the number of endangered avian species in the last years [22] and spillovers of viral strains coming from poultry vaccines have already been reported in wild bird populations [23,24]. By better characterizing the virome of wild birds in remote regions, not only will we expand our knowledge about the global viral diversity, but we will also be able to recognize the potential threats that endangered species might be facing and observe the influence of those viruses on the dynamics, structure and functioning of the ecosystems.

Next generation sequencing (NGS) has been the principal tool for the discovery of novel virus sequences from avian samples, being wild birds the group where they have been mainly described in the last years [22,25]. However, to our knowledge, no metagenomics or metatranscriptomics study has been performed in populations of wild birds from remote regions in the Neotropics and, as mentioned before, the virome of some important avian groups remains virtually unknown even in more studied areas. Therefore, NGS may increase the rate of virus discovery compared to other approaches.

In this study, we used a NGS approach to analyze the cloacal virome of passerine-dominated wild bird communities from two different habitats: a remote and primary rainforest in French Guiana with limited research about avian viruses and a Mediterranean forest in Spain, a region where research about birds and their parasites is more abundant. Our general objective was to show how deep sequencing analyses of samples coming from remote areas and understudied wildlife species can efficiently increase the knowledge of virus diversity by contributing with relevant information about novel viral sequences and viral-host relationships. To this end, we analyzed whether sampling passerine birds (a non-canonical bird taxon in studies of avian viruses) in a remote area (Neotropical rainforest) contributed genetically or functionally more singular viruses than sampling passerines in a more researched area (temperate European forest). We also identified new avian and possibly non-avian viruses carried by wild birds in these two locations. All this information will provide us with important knowledge on virus diversity and virus ecology in pristine areas.

## 2. Materials and Methods 

### 2.1. Sample Collection

A random sampling of understory bird species was carried out in two different sites: the Nouragues Natural Reserve (French Guiana) and La Herrería forest (Spain). The Nouragues Natural Reserve is located in a tropical rainforest in northern South America (4°05′N, 52°40′W), where average temperature is near 26 °C throughout the year and relative humidity is usually high. The climate is very wet in general (annual precipitation > 3000 mm) although there is a dry season with substantially less rainfall between August and November [26]. Birds were mist-netted in Pararé and Inselberg camps in January 2016 (rainy season) and October-November 2016 (dry season). La Herrería is a broadleaved forest located at 900 m.a.s.l. in the center of the Iberian Peninsula (40°34′ N, 4°09′ W) with a continental Mediterranean climate. The average annual precipitation is around 800 mm and there is a dry season between June and September. Although the annual mean temperature is 13 °C, the average monthly temperatures range from 5 °C in January to 23 °C in July [27]. Sampling in La Herrería forest was carried out during the bird breeding season (April–July) of 2018. 

In both locations, the same standardized protocol was followed to prevent differences due to distinct sampling methods. Cloacal samples were collected using sterile swabs (Nerbe Plus), which were preserved in 800 µL of universal viral transport medium (VTM) (Becton Dickinson) and kept frozen until molecular analyses. A total of 406 cloacal samples from 72 bird species were collected in the Nouragues Natural Reserve, and 92 cloacal samples from 20 bird species were obtained in La Herrería. The birds were marked with metal rings to avoid resampling and released unharmed at the site of capture. The different number of species sampled in each site is representative of the different species diversity of Neotropical rainforest and Mediterranean broadleaved forests. All methods were carried out in accordance with European Union and national French and Spanish regulations. Capture, sampling and transport of samples were authorized by the Service of Natural Environments, Biodiversity, Sites and Landscapes, Regional Directorate for the Environment, Planning and Housing at French Guiana (license 030418) and the General Directorate for the Environment of Madrid (license 10/209664.9/18). The experimental protocols were approved by the Committee on Animal Testing of Complutense University (CEA-UCM, authorization number 44-2016).

### 2.2. Sample Selection

For each locality (Nouragues and La Herrería), 50 cloacal samples with abundant fecal matter (to make sure that those samples were properly collected) were selected, including as many different avian species as possible to maximize the number of species analyzed. We grouped them to create five pools of 10 samples in each of the two localities. When more than one individual of the same species were present, they were grouped in the same pool. In total, 32 and 18 different species were selected in Nouragues and La Herrería, respectively. Species of the order Passeriformes accounted for 92% of samples in Nouragues and 94% in La Herrería, other species were small-sized birds sharing the forest undergrowth with passerines (Table S1 and Table S2). In relation to the age of selected birds, 96% (48/50) of individuals from La Herrería were adults while this percentage reached up to 98% (49/50) in Nouragues. Regarding their foraging niche, the vast majority (47 birds) of the 50 individuals sampled in Nouragues Natural Reserve belonged to species that regularly feed on invertebrates (invertivorous or omnivorous). Only two individuals of the species *Dixiphia pipra* (frugivore) and one of the species *Micrastur ruficollis* ssp. *concentricus* (carnivore) belonged to species that do not feed on invertebrates. The totality of the birds analyzed in La Herrería belonged to species that feed on invertebrates (mostly arthropods), especially during the breeding season, when the samples were taken. 

### 2.3. Sample Processing and Next Generation Sequencing

Individual cloacal samples were vortexed, and swabs were squeezed to release epithelial cells before being discarded. The VTM was centrifuged at 13,000 rpm for 1 min to pellet out epithelial cells. Pellets were resuspended in 250 µL of PBS and subjected to 2 freeze-thaw cycles at −80 °C to maximize the release of viral particles and filtered through 0.45 µm pore-sized column filters at 8000 rpm for 5 min. An aliquot (50 µL) of the filtrate of each sample was combined with nine others to make five pools. Each pool was treated with a mixture of nucleases (Turbo DNase, Ambion, Carlsbad, CA, USA; Baseline-ZERO, Epicenter, Madison, WI, USA; Benzonase, Novagen, San Diego, CA, USA; RNAse One, Promega, Fitchburg, WI, USA) to digest unprotected nucleic acids, including host DNA/RNA. Lastly, viral RNA/DNA was extracted with the MagMAX Viral RNA Isolation Kit (Thermo Fisher) according to the manufacturer’s instructions. The extracted viral nucleic acids were subjected to library preparation, after random RT-PCR amplification, by using QIAseq FX DNA Library Kit (Qiagen, Hilden, Germany). Normalized samples were pooled and sequenced using 600-cycle MiSeq Reagent Kits v3 (Illumina, San Diego, CA, USA) on a MiSeq platform. The generated raw reads were first qualitatively checked, trimmed and filtered to remove polyclonal and low-quality reads (<55 bases long) using CLC workbench (Qiagen). The remaining filtered raw reads were de-novo assembled separately using Trinity v2.6.642 [28] and CLC workbench and compared with a non-redundant and viral proteome database using BLASTx with an E-value cut-off of 0.001. The virus-like contigs and singlets were further compared to all protein sequences in non-redundant protein databases with a default E-value cutoff of 0.001. For each library we use unique dual indexes in order to minimize the cross-contamination between the libraries. Furthermore, to remove all possible contamination, a read was presumed to be a contaminant from another library if its abundance compared to other libraries was extremely low and, at the same time, the virus read shared a really high (98–100%) nucleotide sequence identity with a virus from another library. The viral metagenomics output has been visualized and analyzed in MEGAN [29]. The complete dataset from the cloacal deep sequencing analyses has been deposited in the NCBI’S Sequence Read Archive (accession numbers PRJNA669430 and PRJNA669438).

### 2.4. Detection of Individuals Positive for the Viruses

The birds carrying the viruses of interest were detected by RT-PCR using Verso 1 step RT PCR kit (Thermo Fisher Scientific, Somerset, NJ, USA) following manufacturer’s instructions and a specific set of primers we designed for each virus (Table S3). First we carried out the PCR/RT-PCR using DNA/RNA extractions of the pools as a template and, once we knew the positive pool, we repeated the PCR/RT-PCR with the individual extractions. We visualized the PCR/RT-PCR product in a 2% agarose gel stained with GelRed^®^ 100×.

### 2.5. Genomic Analysis of the Novel Viruses

Genome sequence analysis and genomic organization were performed using Geneious v11 (Biomatters, Auckland, New Zealand), EditSeq and SeqMan tools of the DNASTAR 5.0 software package (DNASTAR, Madison, WI), and ORFfinder (https://www.ncbi.nlm.nih.gov/orffinder/). Sequences of the putative proteins of the novel viruses were analyzed using InterPro (http://www.ebi.ac.uk/inter pro/) and Motif Scan (https://myhits.sib.swiss/cgi-bin/motif_scan) software to find conserved motifs. Similarity and possible recombination events along the amino acid sequence of ORF1 of the different members of the *Hepeviridae* family were examined using RAT (https://omictools.com/rat-tool) and the region beyond the polymerase of unknown function was analyzed using Phyre2 in search of similarity to known protein motifs [30]. In this study, we include data of the complete genomes of four different astroviruses (Passerine astrovirus-1-4; PasAstV-1-4) and a novel gyrovirus (gyrovirus 11; GyV11) that have been fully described elsewhere [31,32], as they were retrieved from these metagenomic analyses.

### 2.6. Phylogenetic and Taxonomic Analysis

Multiple alignment of amino acid sequences were carried out in MUSCLE [33] (https://www.ebi.ac.uk/Tools/msa/muscle/). Distance matrices, best-fit nucleotide substitution model tests and maximum likelihood phylogenetic trees were inferred using MegaX [34].

### 2.7. Comparative Analysis

We compared the similarity of the contigs obtained in Nouragues and La Herrería with reference sequences in databases to test if this similarity was lower in Nouragues due to its remote location. First, contigs obtained with MEGAN in Nouragues and La Herrería were aligned to reference sequences in Genbank using Blastn and Blastx (https://blast.ncbi.nlm.nih.gov/Blast.cgi). Additionally, we classified the putative host (animal, plant, prokaryote, etc.) of the viral contigs based on the host of the closest homologue in Blast showed by MEGAN. We used Blastn and Blastx to hint into the genetic novelty (as scored by divergence in nucleotide sequence) and the putatively functional novelty (divergence in the encoded protein sequence) of newly discovered viruses, respectively. We blasted all contigs we obtained and we used only those which showed similarity to virus sequences both in Blastn and Blastx for the statistical analyses. Before the statistical analysis, we removed all hits showing similarity to previously published viruses from our study (GyV11 and PasAst-V-1-4). We used bit scores and identity values as two variables indicative of how similar the contig is to reference sequences in databases (the higher the bit score, the lower the probability of an alignment by chance; and the higher the identity value, the more similar to reference sequence). Variation in bit scores and identity percentages of these alignments between sampling locations were analyzed using generalized linear mixed models with normally distributed errors in R v 4.0.2 [35]. In these models, site was included as a fixed factor and virus family was a random factor. Alignment length was included as a fixed covariate when identity percentage was the response variable to control for different alignment lengths. All variables were z-standardized to bring them to the same scale prior to the analyses.

The Shannon–Weaver index (*H*) was calculated for each location as a measure of viral richness (alpha diversity), equitability of the different viral families was calculated using Pielou’s index (*J*) and the difference in viral community composition was estimated by Jaccard distance using the package vegan [36].

## 3. Results

### 3.1. Composition of Nouragues/La Herrería Cloacal Viromes

**Figure 1:**
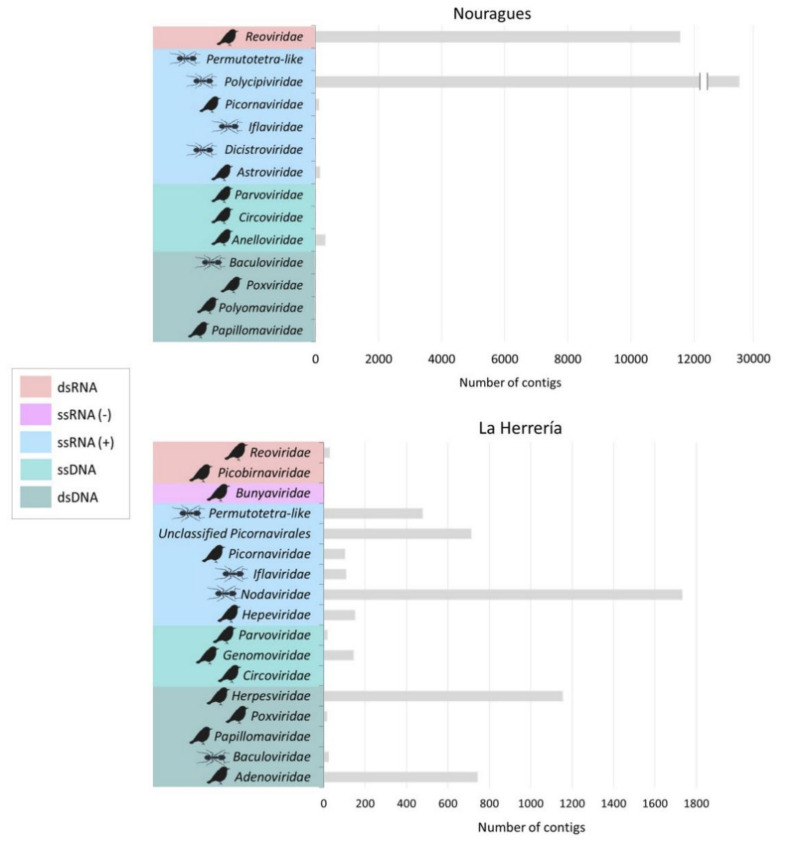
Number of contigs showing similarity to viruses of families infecting animals sequenced from cloacal samples of wild birds from Nouragues (French Guiana) and La Herrería forest (Spain). The colors indicate the type of genome of each family. The ant silhouette highlights those viral families infecting invertebrates. The bird silhouette highlights viral families with reported avian viruses.

In Nouragues (French Guiana), we obtained a total of 1,888,004 reads, 0.53% of them showing similarity to viruses and 7.88% to other known organisms, while in La Herrería (Spain), we obtained a total of 2,919,868 reads, 0.27% of them showing similarity to viral sequences and 21.65% to other known organisms. Regarding viral contigs, RNA viruses were the predominant group in both localities representing 90.9% in Nouragues and 83.5% in La Herrería. However, we recovered a low number of viral contigs showing similarity to ssRNA (−). DNA viruses were less abundant than RNA viruses (4.8% in Nouragues and 15.7% in La Herrería) and contigs showing similarity to unclassified viruses were a minority in both locations. The great majority of the putative viral contigs in both locations showed similarity to animal viruses. In Nouragues, the families *Polycipiviridae* and *Reoviridae* represented, by far, the main families of animal viruses (70.3% and 28.1% respectively; Figure 1). Nevertheless, sequences from the families *Astroviridae*, *Anelloviridae* and *Picornaviridae* were also present and we were able to obtain the complete genomes of four different astroviruses, a novel gyrovirus (these viruses have been fully described elsewhere, see Fernandez-Correa et al. 2019 and Truchado et al. 2019 [31,32]) and a novel picornavirus (Table 1). Identity values were relatively high in general for contigs of putative avian viruses in Nouragues (>60%; Table 1) but these identities were lower when analyzing complete genomes.

Viral richness was higher in La Herrería (*H* = 2.21) than in Nouragues (*H* = 1.28). Moreover, viral families of La Herrería were more equitably distributed (*J* = 0.59) than viral families of Nouragues (*J* = 0.36). Viral composition similarity between Nouragues and La Herrería was low (Jaccard index = 0.38).

In La Herrería, the percentage of contigs with similarity to animal viruses was very close to Nouragues (94%). However, the second most relevant group were plant viruses (2.7%) followed closely by other eukaryotic viruses (2.6%). The number of different virus families of contigs showing similarity to animal viruses was slightly higher in La Herrería than in Nouragues, most of them being ssRNA (+) or dsDNA viruses (Figure 1). Additionally, the proportion of contigs belonging to virus families exclusively infecting vertebrates in La Herrería was higher than in Nouragues, where contigs of viruses of invertebrates prevailed. Among ssRNA (+) animal viruses, the greater part of the contigs showed similarity to viruses likely coming from invertebrate hosts. Fewer different contigs of putative avian virus were obtained in La Herrería deep sequencing and no complete genome could be assembled (Table 2).

**Figure 2:**
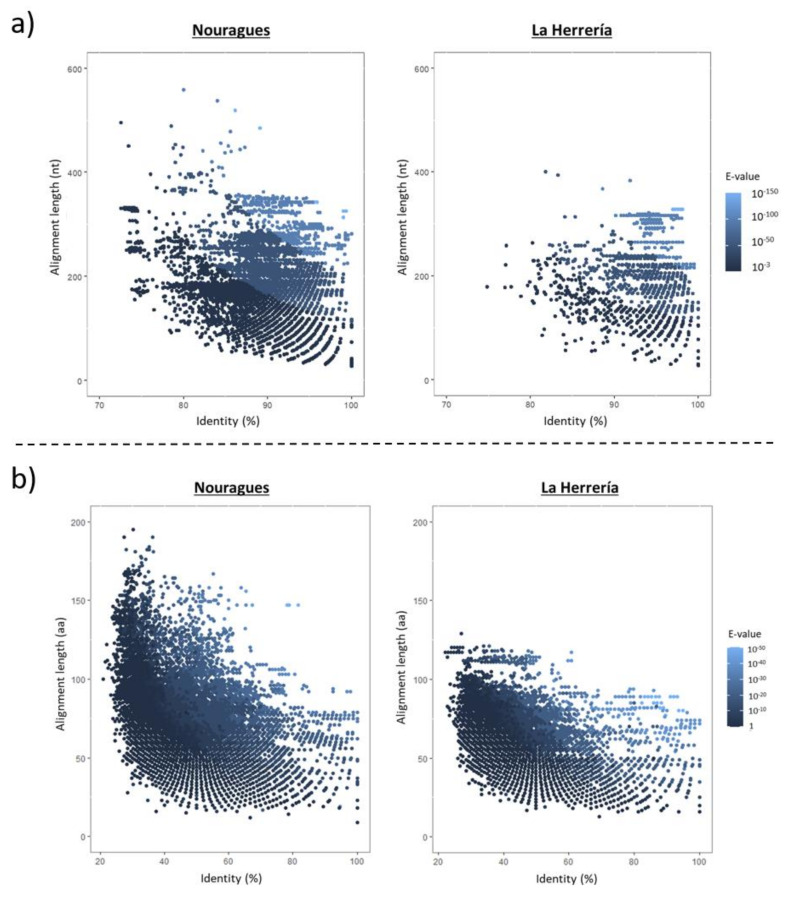
Alignment length (nt, nucleotides; aa, amino acids) vs identity percentage for the contigs obtained in Nouragues and La Herrería cloacal deep sequencing analysis when compared to reference sequences in GenBank using Blastn (**a**) and Blastx (**b**). Each dot is colored according to the E-value of the alignment in order to increase the descriptive value of the graph.

**Figure 3:**
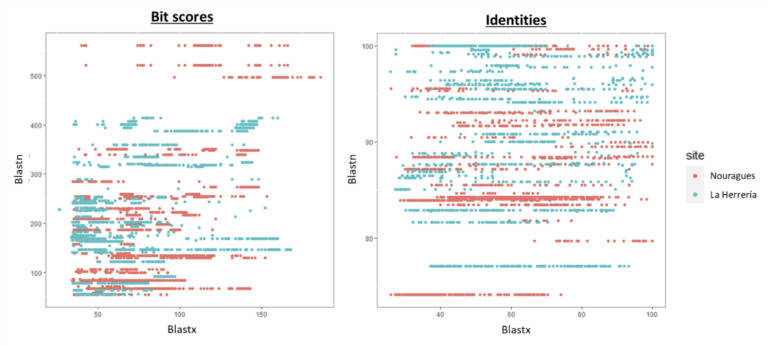
Comparison between values obtained in Blastn and Blastx for bit scores and identity percentages for a given contig in Nouragues and in La Herrería.

**Figure 4:**
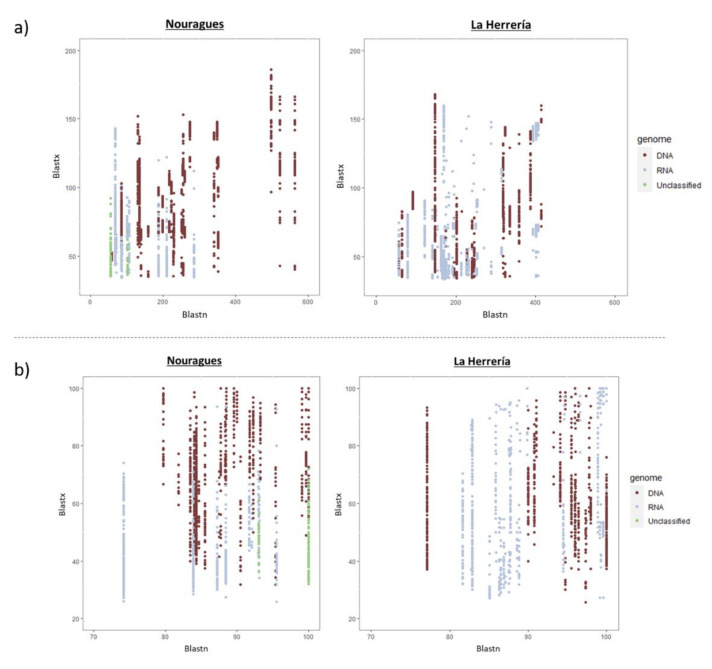
Comparison between values of bit scores (**a**) and identities (**b**) of contigs sequenced in Nouragues and La Herrería using Blastn and Blastx considering the type of genome of the novel viruses. The type of genome (DNA, RNA or unclassified) was obtained from the viral group each contig was assigned to by the software MEGAN.

When comparing the total number of viral reads in both locations to reference sequences in databases, we observed that there were no reads with long alignment lengths and low similarity values in La Herrería using Blastn or Blastx, as it happened in Nouragues (Figure 2). Bit scores and identity percentages were significantly lower in Nouragues than in La Herrería using Blastn (Table 3; Figure 3). However, bit scores and identity values were significantly lower in La Herrería using Blastx. When we compared the values obtained in Blastn and Blastx of these two variables for a given contig, we observed that unclassified viruses and a great part of RNA viruses in Nouragues showed the lowest values of bit scores both in Blastn and Blastx (Figure 4). A similar result was obtained for La Herrería. Results for identity values are less clear. The majority of RNA viruses showed lower values in Blastn and Blastx in comparison to DNA viruses. Unclassified viruses of Nouragues showed high identity values in Blastn but low values in Blastx, what could be explained as alignments by chance of short contigs to reference nucleotide sequences (Figure 4). 

### 3.2. Novel Viruses Found in Nouragues/La Herrería

**Figure A1:**
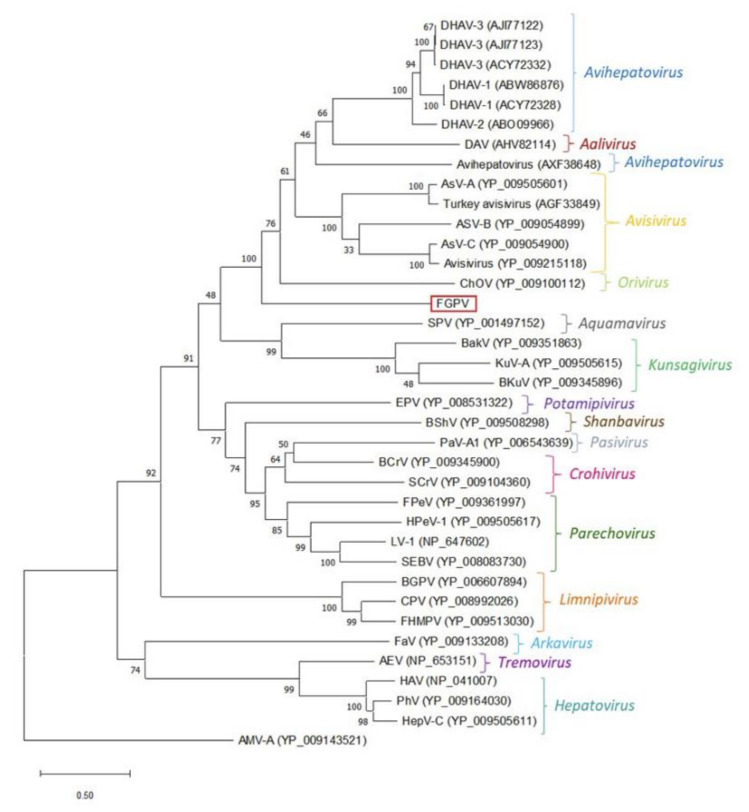
Maximum likelihood tree based on amino acid sequences of P1 region of the family *Picornaviridae* using LG + G + I + F method (bootstrap value = 1000 replicates). The different genera within the family are indicated to the right of the tree. Taxon information includes species names and GenBank accession numbers. Branch lengths measure the number of substitutions per site. *Ampivirus A* (AMV A) was used as outgroup. AEV, avian encephalomyelitis virus; AsV, avisivirus; BakV, bakunsavirus; BCrV, bat crohivirus; BGPV, bluegill picornavirus; BKuV, bat kunsagivirus; BShV, bat picornavirus; ChOV, chicken orivirus; CPV, carp picornavirus; DAV, duck aalivirus; DHAV, duck hepatitis A virus; EPV, eel picornavirus; FaV, falcovirus; FGPV, French Guiana picornavirus; FHMPV, fathead minnow picornavirus; FPeV, ferret parechovirus; HAV, hepatitis A virus; HepV-C, hepatovirus C; HPeV, human parechovirus; KuV, kunsagivirus; LV, ljunganvirus; PhV, phopivirus; PaV, pasivirus; SCrV, shrew crohivirus; SEBV, Sebokele virus; SPV, seal picornavirus.

We obtained two complete genomes (French Guiana Picornavirus and French Guiana Hepevirus) and two almost complete genomes (French Guiana Reovirus and La Herrería Hepevirus) of novel viruses carried by the wild birds analyzed. In Nouragues, a novel picornavirus provisionally named French Guiana Picornavirus (FGPV; Genbank accession number MT792642) was detected in the cloacal sample of a Rufous-throated Antbird (*Gymnopithys rufigula*). Phylogenetic analysis place FGPV in a divergent branch sister to the genera *Avihepatovirus* and *Avisivirus* (Figure A1). Added to FGPV are the four novel astroviruses and a novel gyrovirus detected in these deep sequencing analyses that were published elsewhere [31,32]. 

**Figure A3:**
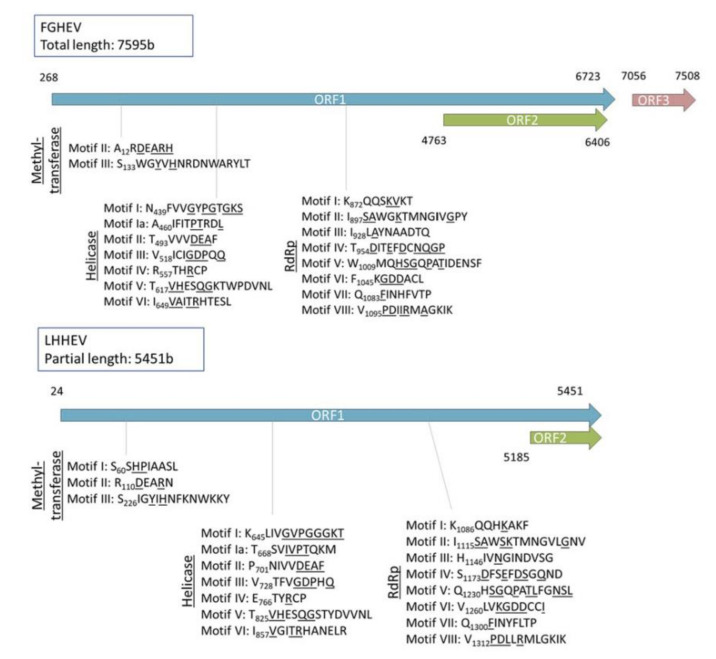
Putative methyl-transferase, helicase and RNA-dependant-RNA polymerase (RdRp) motifs within the ORF1 of French Guiana hepevirus (FGHEV) and La Herrería hepevirus (LHEHV). Conserved amino acids coincident with those describe by Koonin et al. (1992) [72] are underlined.

At the same time, three other viruses stood out because they were highly divergent to their closest relatives, although they cannot be clearly classified as avian viruses. A novel reovirus, provisionally named French Guiana Reovirus (FGRV; Genbank accession numbers MT792643-48), was detected in the cloacal sample of a Wedge-billed Woodcreeper (*Glyphorynchus spirurus*) in Nouragues. The phylogenetic tree using the amino acid sequences of the putative RNA-dependent RNA polymerase showed that this novel reovirus is grouped with Cimodo virus in a divergent clade within the subfamily *Spinareovirinae* (Figure A3). Finally, two hepe-like viruses were detected in the cloaca of two invertivore birds: a plain xenops (*Xenops minutus*) in Nouragues (provisionally named French Guiana Hepevirus; FGHEV) and a European Robin (*Erithacus rubecula*) in La Herrería (provisionally named La Herrería Hepevirus; LHHEV). Although only FGHEV is fully sequenced, both showed unusual arrangements in their genome architectures. Phylogenetic analyses placed LHHEV as a clade within hepe-like viruses, but the phylogenetic location of FGHEV was less clear (Figure S8). The complete genome of FGHEV and the partial genome of LHHEV were deposited in Genbank under accession numbers MT792641 and MW147021 respectively. A more detailed characterization of the viruses can be found in the Appendix A.

## 4. Discussion

In a context where viral discovery and surveillance in wildlife have become one of the main goals to prevent disease outbreaks and global pandemics [1,37], this study adds information about novel viruses harbored by birds in wild populations and potential gaps of knowledge of the global virus diversity associated with the paucity of research in understudied species and geographic regions. Using a discovery-driven approach to uncovering virus diversity, we have examined the cloacal virome of passerine-dominated bird communities sampled in a tropical rainforest and in a Mediterranean habitat, in order to reveal the importance of remote areas and wildlife as sources of relevant new information in the field of virology. We are aware that our comparison of virus singularity in samples from remote and canonical sampling regions would be much more insightful if we could replicate it to observe the same pattern in independent samples. Additionally, the diversity of viruses we found could be influenced by the random amplification method we followed as pretreatment. However, as a first approach to the importance of this discovery-driven perspective our data provide valuable insights into the advantages of exploring the unexplored to boost up biological discovery. Additionally, we shed some light on the cloacal virome of wild passerines, addressing the issue for the first time despite the relevance and ubiquity of this group of birds. We discovered several novel and divergent viruses of the families *Anelloviridae*, *Astroviridae*, *Hepeviridae*, *Picornaviridae* and *Reoviridae*. The novel viruses of the families *Anelloviridae* and *Astroviridae* were described in detail elsewhere [31,32].

### 4.1. Composition of Nouragues/La Herrería Cloacal Viromes

The presence of contigs showing little or no similarity to reference sequences highlights the need for further research in the virome of birds and other wildlife. We obtained a high number of reads showing no similarity with sequences in the GenBank database. As this proportion of reads with unknown origin is very similar in Nouragues and La Herrería, this result could be explained mainly because wild birds, and especially wild passerines, have remained understudied in this type of analyses compared to other avian groups such as poultry or waterfowl. It is considered that up to 90% of viral reads in deep sequencing analyses can be considered as “viral dark matter” [38] as they do not align to any available viral sequence, especially when dealing with highly divergent viruses and short fragments. Additionally, research in virology has been highly biased towards human and other mammalian viruses [38,39], so analyzing the virome of non-canonical hosts represents a challenge as closely related sequences are scarce or absent in public databases. Thus, the majority of the contigs sequenced in our study could not be assigned to any known taxon, showing the importance of continuing studying unexplored species and regions to expand the available reference sequences in the future. 

Regarding viral reads, RNA viruses were the predominant group in both sampling sites. RNA viruses have been shown to be the most abundant group in other deep sequencing analysis from avian fecal samples [16,40,41]. However, not all RNA viruses were equally represented. It is remarkable the low number of reads related to ssRNA (–) viruses we obtained in both Nouragues and La Herrería. In some studies of fecal avian virome, ssRNA (–) are not very abundant but are present to some extent [40] while in others they are completely absent or not highlighted by the authors [16,20,41,42]. Avian influenza virus, the main ssRNA (–) viruses found in birds so far, has been shown to be present in fecal samples of waterfowl, turkeys and chickens, being wild aquatic birds their main reservoir [43,44,45]. However, avian influenza virus is not present in all bird groups. Wild passerines do not seem to play a role in the transmission of avian influenza virus [46]. As passerines represent the great majority of the birds sampled in this study and we did not collect samples from aquatic birds, this could be an explanation of the low number of reads related to ssRNA (–) viruses we obtained. On the other hand, Rosseel et al. (2015) showed that different pre-treatments of samples before deep sequencing had different effects on the detection of ssRNA (–) viral reads [47]. More specifically, a random PCR amplification before deep sequencing had a negative impact on the number of ssRNA (–) detection. As our samples were pre-treated this way, it is possible that the number of reads related to ssRNA (–) viruses was underrepresented in our study. In relation to DNA viruses, they were a minority in both locations, representing 15.7% of viral reads in La Herrería and only 4.1% in Nouragues. These results differ from other studies of fecal virome in birds, where this percentage is much higher [16,17,41]. Only Zhao et al. (2018) obtained a similar proportion of DNA virus reads in their study with the fecal virome of Jinding ducks although they followed a different pretreatment [18].

The relatively low value of similarity in viral composition of both viromes (38%) reveals that, even though we were sampling passerine-dominated communities, the viral families we obtained from the two locations were different in great part. This could be due to different host composition or time of the year but could be also due to different locations.

Viral classification by host showed that animal viruses represented the greatest proportion in our study. In Nouragues, prokaryotic viruses were the second most abundant group, in contrast with the results in La Herrería, where plant and other eukaryotic viruses were more abundant. The majority of animal viruses we found had similarity with viruses of invertebrates, as expected given the type of sample and the diet of the birds analyzed. Sequences of insect viruses were also abundant in previous deep sequencing analyses of insectivorous animals such as birds [16,41,48] or bats [49,50], also reflecting their dietary preferences. The remarkably high percentage (60.8%) of contigs showing similarity to the *Polycipiviridae* family (arthropod viruses) in Nouragues cloacal virome could be explained because of the great number of invertivore species among the birds sampled, being an example of how the virome can be influenced by the structure and ecology of host community, as it was shown in other wild bird populations [10]. However, a divergent member of this family has recently been reported in the stool of a frugivorous bat, being the first time that these viruses appear in a vertebrate [51]. Although the presence of viruses of the *Polycipiviridae* family in cloacal or rectal samples seems likely due to diet, the possibility that this newly described viral family has members infecting vertebrate hosts cannot be ruled out. 

Our discovery-driven approach to documenting virus diversity showed that the endeavor of uncovering new avian viruses may not only benefit from studies of non-canonical host species, but also from sampling remote areas seldom explored in virus research. Viral contigs found in Nouragues showed, in general, significantly lower bit scores and identity percentages using Blastn. This would imply that nucleotide sequences of viruses present in the cloaca of wild birds in remote regions are less similar to known viruses than the viruses carried by wild birds living in more studied ecosystems. Furthermore, although we found higher diversity of virus families in La Herrería, the singularity of these viruses was lower compared to Nouragues, where all complete or almost complete viral genomes were very divergent to their closest relatives in the phylogenetic analyses. On the contrary, bit scores and identities were, in general, significantly higher in Nouragues than in La Herrería, suggesting that amino acid sequences of viruses from Nouragues are more similar to those in databases. This apparently contradiction could reflect the difference between genetic singularity (nucleotide sequences) and functional singularity (amino acid sequences). Nucleotide sequences in Nouragues are less similar to reference sequences probably due to the singularity of the sampling area and carriers, very different from the traditional ones. Moreover, the isolation of Nouragues ecosystem implies more viruses with unknown nucleotide sequences of which we detect only their functionality through their amino acid sequences using Blastx. Additionally, contigs showing similarity to unclassified viruses in Nouragues showed high identity values in Blastn but, however, low bit scores which could reflect alignments by chance of short viral reads to reference sequences. This would explain the high similarity observed between these contigs and reference nucleotide sequences (Blastn) that is not translated into the correspondent putative proteins available in Genbank database (Blastx). Taking all this into consideration, we can suggest that wild birds of Nouragues carry genetically more singular viruses belonging to fewer virus families than wild birds of La Herrería. However, this result does not seem to be true for functional singularity of the viruses as it is captured from protein sequence divergence, so more comparative studies in remote regions are needed considering both types of singularity in order to clarify this trend. 

### 4.2. Novel Viruses of Interest Found in Nouragues/La Herrería

Focusing on viruses of vertebrates, the main families we found in our study have been frequently found in other fecal viromes of birds. For example, picornaviruses are usually found in this type of samples, irrespective of whether birds are domestic or free-living or if they are healthy or not [16,41,45,52,53,54]. Astrovirus sequences also have been frequently reported in previous metagenomics analysis of avian fecal samples [41,53,54,55]. However, the four novel astrovirus genomes we obtained from wild birds from Nouragues constitute a putative new species, providing important new information in relation to the family *Astroviridae* [31]. The same happens with GyV11, the divergent novel gyrovirus we found in the same set of samples [32]. The four astroviruses detected in Nouragues and GyV11 are examples of divergent viruses related to important avian pathogens involved in intestinal disorders and so far unknown circulating in a remote region. Nevertheless, they were not the only putative avian viruses whose complete genome was obtained in this population of Neotropical birds. FGPV was present in the cloacal sample of a Rufous-throated Antbird, being the first time that a picornavirus is detected in a bird of the family Thamnophilidae. The family *Picornaviridae* is the most diverse among ssRNA (+), with more than 75 accepted species infecting mainly mammals and birds [56]. However, the discovery of a novel, divergent picornavirus likely corresponding to a new species infecting birds shows that there is an unknown diversity of this group of viruses yet to be discovered. In fact, novel and divergent picornaviruses have been recently detected in hosts rarely sampled before [45,57,58] and, prior to these studies, only five out of 18 species of avian picornaviruses had been described in wild birds [48,59,60,61,62]. It is interesting that the Rufous-throated Antbird positive to FGPV was also positive for astrovirus, showing a possible coinfection. Unfortunately, as picornavirus infections are usually asymptomatic, it is difficult to determine the effect of FGPV, as in the case of other novel members of the family.

Regarding FGRV and the two novel hepe-like viruses we have detected in our samples, we cannot clearly classify them as avian viruses or as viruses of invertebrates that were in the diet of the analyzed birds. Reoviruses are dsRNA viruses infecting a wide variety of hosts and causing gastroenteritis and respiratory diseases in vertebrates. The closest reovirus to FGRV is Cimodo virus, detected in Africa and which likely infects mosquitoes [63]. Both reoviruses appear to form a new genus within the subfamily *Spinareovirinae*. FGRV was present in the cloacal sample of a Wedge-billed Woodcreeper (*Glyphorynchus spirurus*), an insectivorous bird of Nouragues, so it is possible that this novel reovirus was infecting insects that this bird had fed on. However, we cannot discard that FGRV and Cimodo virus might be arboviruses transmitted by mosquitoes (or other arthropods) to birds, as neither of them have been tested to infect bird cell cultures [63].

A similar situation occurs in the case of the two novel hepe-like viruses we detected: FGHEV and LHHEV. Hepeviruses are important zoonotic viruses causing hepatitis E and splenomegaly with high mortality rates among vertebrates. Hepe-like viruses are a group phylogenetically related to hepeviruses recently described whose effect on their host is still unknown [64]. Our results suggest that FGHEV and LHHEV would belong to this latter group for several reasons. Firstly, their ORF arrangement and genome length are more similar to those of the hepe-like viruses than to those of hepeviruses [65]. Phylogenetic analysis supports this hypothesis in the case of LHHEV, placing it clearly within the hepe-like group, whose members have been mainly detected in invertebrates [64,66,67] or in fecal samples of animals feeding on them [68,69,70]. Nonetheless, the phylogenetic position of FGHEV is more ambiguous, as it could be related to hepe-like viruses of invertebrates or to hepeviruses of vertebrates depending on the fragment selected to infer the tree. Given that the species of birds carrying both novel hepe-like viruses feed on invertebrates, both FGHEV and LHHEV could be actually viruses of invertebrates that were part of the diet of the birds. However, the fact that we were able to sequence the whole genome of FGHEV and almost the complete genome of LHEEV, argues in favor that both viruses could have maintained their integrity until the end of the avian digestive tract. In that case, birds would act as dispersers of these viruses and their putative invertebrate hosts could become infected if they got in close contact with bird droppings. This transmission route through the feces of predators has been shown to happen for a virus infecting the gypsy moth [71] and could be used also by the aforementioned FGRV and *Polycipiviridae* virus in Nouragues, as both groups were the most abundant in those cloacal samples. Unfortunately, little is known yet about the pathogenicity and ecology of the hepe-like group. Therefore, we cannot rule out that FGHEV and LHHEV are exclusively novel avian pathogens or that they could infect and circulate among both invertebrate and vertebrate species, especially FGHEV. In any case, these unclear results for FGHEV are the evidence that it belongs to a yet unknown diversity of viruses circulating in this remote area, so further research is needed to clarify its ecology, epidemiology and its closer relatives in the phylogeny.

## 5. Conclusions

In general, our research reveals how extending the focus to non-canonical hosts and regions is crucial for viral discovery. Most of the potential avian viruses we obtained using a discovery-driven approach were different enough to be considered novel species or even genera. This seems to be mainly the effect of the limited existing research on the virome of wild birds and, especially, of wild passerines. Therefore, carrying out deep sequencing analyses in bird species other than poultry and waterfowl contributes substantially to gain better insight into avian virology. In fact, none of the novel viruses described in birds during recent years were detected in poultry [22]. Moreover, our results also show how remote regions harbor an unknown diversity of viruses that is yet to be described, and how preserving and studying pristine forests is highly relevant in the research of emerging infectious diseases to avoid future spillovers that affect humanity and biodiversity. Thus, a good approach to widen the knowledge about animal viruses in general would be to combine sampling in understudied animal hosts with analyzing the virome of wild animals in remote regions.

## Figures and Tables

**Table 1:** Contigs and complete genomes obtained by deep sequencing from cloacal samples of wild birds from Nouragues showing similarity to putative avian viruses. Closest homologs, identity and coverage values for partial genomes were obtained using Blastx.

Group	Family	Length (b)	Virus Name (or Closest Homolog)	Accession	Genome Region	Identity	Query Cover
dsDNA	*Papillomaviridae*	407	Francolinus leucoscepus papillomavirus 1	YP_003104804	L1	79%	99%
	*Poxviridae*	176	Fowlpox virus	AAZ14082	39 kDa core protein	100%	63%
ssDNA	*Circoviridae*	1955	Canary circovirus	NP_573442	Replicase	67%	24%
	*Parvoviridae*	346	Goose parvovirus	ABD76400	Nucleocapsid protein	61%	76%
	*Anelloviridae*	2138	GyV11	MH638372	Complete genome	-	-
ssRNA (+)	*Astroviridae*	6745	PasAstV-1	MK096773	Complete genome	-	-
		6864	PasAstV-2	MK096774	Complete genome	-	-
		6628	PasAstV-3	MK096775	Complete genome	-	-
		6870	PasAstV-4	MK096776	Complete genome	-	-
	*Picornaviridae*	7645	FGPV		Complete genome	-	-

**Table 2:** Contigs obtained by deep sequencing from cloacal samples of wild birds from La Herrería showing similarity to putative avian viruses. Closest homologs, identity and coverage values were obtained using Blastx.

Group	Family	Contig Length (b)	Closest Homolog(s)	Accession	Genome Region	Identity	Query Cover
ssDNA	*Poxviridae*	903	Shearwaterpox virus	ARE67299	Immunoglobulin domain	45%	27%
	*Parvoviridae*	472	Dependoparvovirus	QHY93491	Non-structural protein	86%	41%
			Parus major densovirus	YP_009310052	ORF5	50%	41%
ds RNA	*Reoviridae*	446	Rotavirus B	ANN82201	RdRp	51%	71%
		283	Rotavirus G	AXF38053	NSP2	47%	63%
		236	Rotavirus J	APQ41756	VP4	63%	54%

**Table 3:** Summary of the general linear mixed models analyzing bit scores and identities of the alignments between contigs sequenced in Nouragues and La Herrería and reference sequences in Genbank database using Blastn and Blastx.

Blastn						
	Bit Scores			Identities		
Predictors	Incidence Rate Ratios	CI	*p*	Estimates	CI	*p*
(Intercept)	121.19	98.73–148.76	<0.001	86.09	84.19–88.00	<0.001
site (Herreria)	1.49	1.41–1.58	<0.001	4.37	3.75–4.99	<0.001
**Random Effects**						
σ^2^	0.13			8.60		
τ_00_	0.30 _family_			17.29 _alignment_length_		
				22.97 _family_		
ICC	0.69			0.82		
N	28 _family_			28 _family_		
				367 _alignment_length_		
Observations	36,197			36,197		
Marginal R^2^/Conditional R^2^	0.038/0.701			0.040/0.831		
**Blastx**						
	**Bit Scores**			**Identities**		
Predictors	Estimates	CI	*p*	Estimates	CI	*p*
(Intercept)	0.65	0.41–0.90	<0.001	0.03	−0.24–0.31	0.819
site (Herreria)	−0.65	−0.67–−0.62	<0.001	−0.27	−0.30–−0.25	<0.001
**Random Effects**						
σ^2^	0.76			0.48		
τ_00_	0.58_family_			0.65_alignment_length_		
				0.57_family_		
ICC	0.43			0.72		
N	38_family_			38_family_		
				170_alignment_length_		
Observations	112,176			112,176		
Marginal R^2^/Conditional R^2^	0.072/0.475			0.011/0.720		

**Figure A2:**
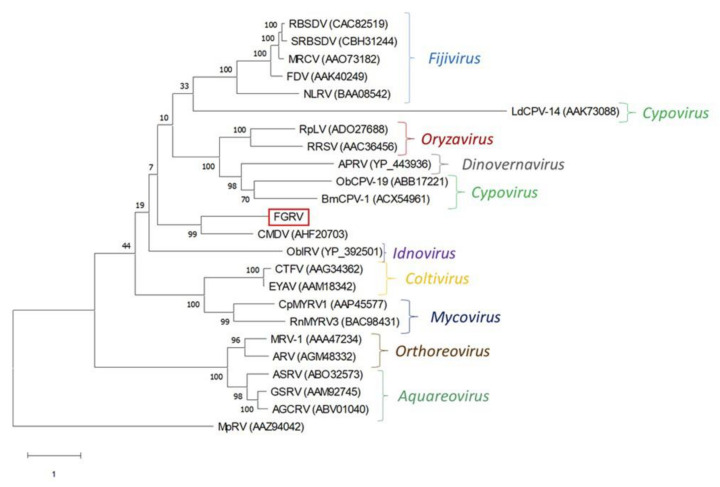
Maximum likelihood tree based on amino acid sequences of RNA-dependent RNA polymerase using LG + G + F method (bootstrap value = 1000 replicates) showing the position of French Guiana reovirus (FGRV) within the subfamily *Spinareovirinae*. The different genera within the family are indicated to the right of the tree. Taxon information includes species names and GenBank accession numbers. Branch lengths measure the number of substitutions per site. Micromonas pusilla reovirus (MpRV), a representative of the subfamily *Sedoreovirinae*, was used as outgroup. AGCR, American grass carp reovirus; APRV, Aedes pseudoscutellaris reovirus; ARV, avian orthoreovirus; ASRV, Atlantic salmon reovirus; BmCPV, Bombyx mori cypovirus; CpMYRV, Cryphonectria parasitica mycoreovirus; CTFV, Colorado tick fever virus; CMDV, Cimodo virus; EYAV, Eyach virus; FDV, Fiji disease virus; GSRV, golden shiner reovirus; LdCPV, Lymantria dispar cypovirus; MRCV, mal de Rio Cuarto virus; MRV, mammalian orthoreovirus; NLRV, Nilaparvata lugens reovirus; ObCPV, Operophtera brumata cypovirus; ObIRV, Operophtera brumata idnoreovirus; RBSDV, rice black streaked dwarf virus; RnMYRV, Rosellinia necatrix mycoreovirus; RpLV, raspberry latent virus; RRSV, rice ragged stunt virus; SRBSDV, southern rice black streaked dwarf virus.

**Figure A4:**
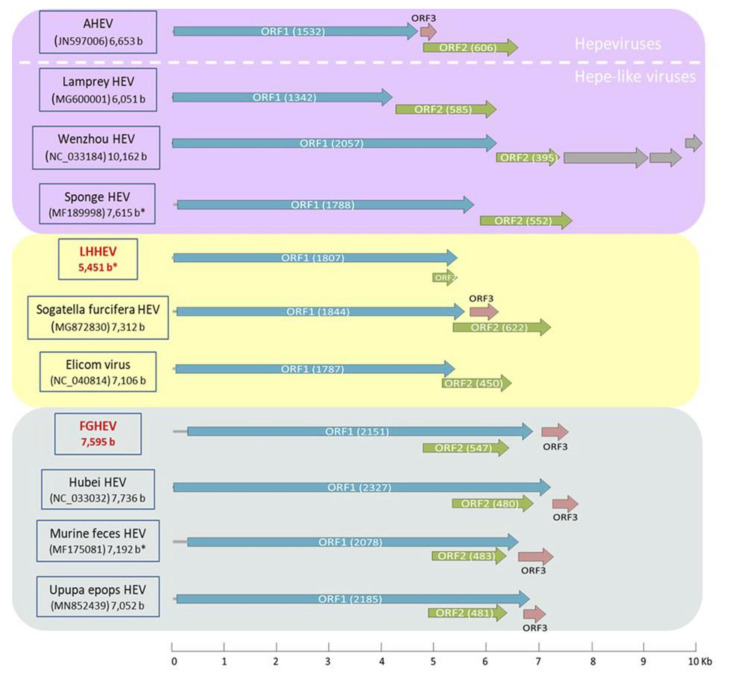
Schematic representation of the genomic organization of French Guiana hepevirus (FGHEV), La Herrería hepevirus (LHHEV) and other hepe-like viruses. Avian hepatitis E virus (AHEV) has been selected as a model of typical ORF arrangement in the family *Hepeviridae*. The genomes have been grouped depending on the position of ORF2: separated from ORF1 as in hepeviruses (violet), partially overlapping with ORF1 (yellow) and embedded within ORF1 (blue). The number in parenthesis indicates the lenght of the ORF and the 5’UTR is represented as a grey segment at the beginning of the genome. The grey arrows of Wenzhou HEV represent proteins of unknown function. Asterisks mark incomplete genomes. The novel hepe-like viruses from this study are highlighted in red.

**Figure A5:**
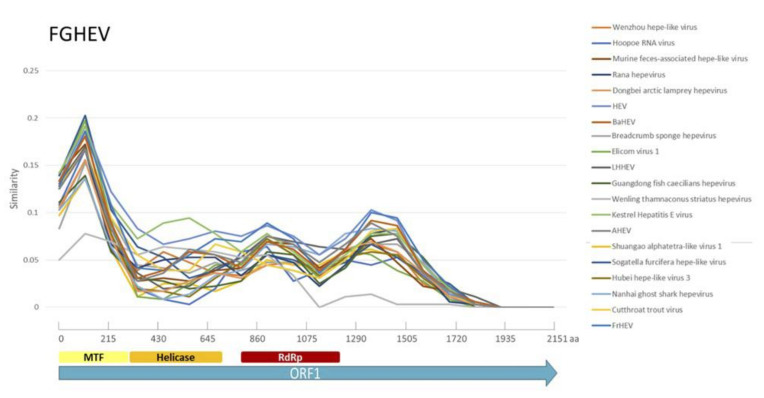
Similarity analysis along the ORF1 amino acid sequence of FGHEV and other members of the family *Hepeviridae* using Recombination Analysis Tool (RAT). Color boxes indicate the conserved motifs found in the FGHEV sequence: methyltransferase (MTF), helicase and RNA-dependent RNA polymerase (RdRp).

**Figure A6:**
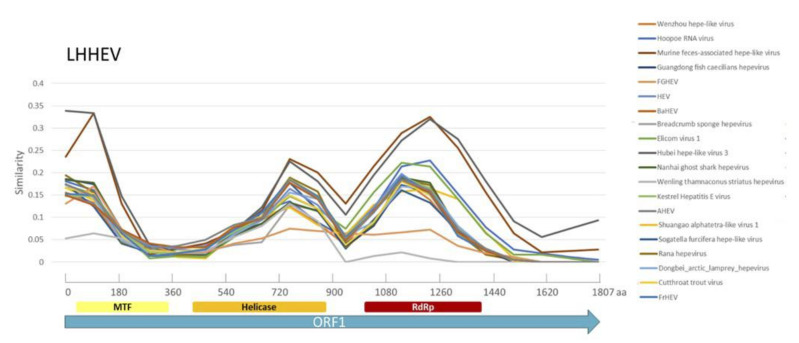
Similarity analysis along the ORF1 amino acid sequence of LHHEV and other members of the family *Hepeviridae* using Recombination Analysis Tool (RAT). Color boxes indicate the conserved motifs found in the LHHEV sequence: methyltransferase (MTF), helicase and RNA-dependent RNA polymerase (RdRp).

**Figure A7:**
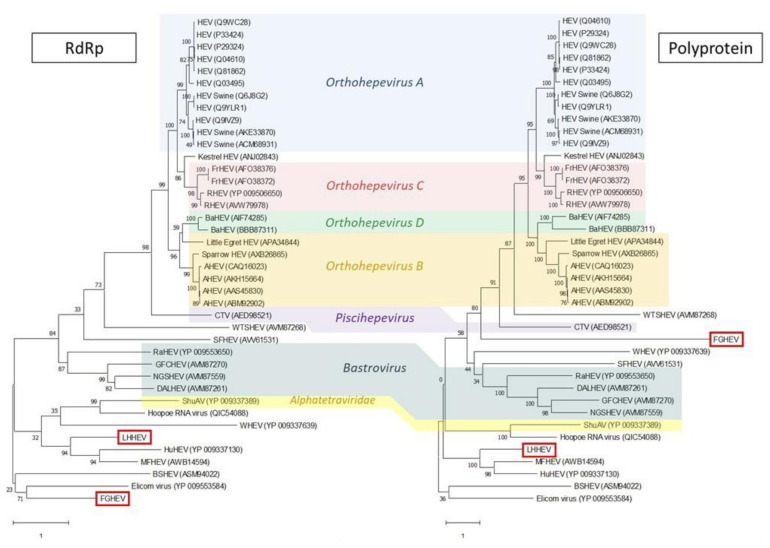
Maximum likelihood trees based on amino acid sequences of RNA-dependent-RNA polymerase (RdRp) and the whole ORF1 (polyprotein) of the family *Hepeviridae* using LG + G + I and LG + G methods respectively (bootstrap value = 1000 replicates). The different genera within the family are highlighted in different colored boxes. Taxon information includes species names and GenBank accession numbers. Branch lengths measure the number of substitutions per site. AHEV, avian hepatitis E virus; BaHEV, bat hepatitis E virus; BHEV, Barns Ness breadcrumb sponge hepe-like virus 1; CTV, Cutthroat trout virus; DALHEV, Dongbei arctic lamprey hepevirus; FGHEV, French Guiana hepevirus; FrHEV, ferret hepatitis E virus; GFCHEV, Guangdong fish caecilians hepevirus; HEV, human hepatitis E virus; HuHEV, Hubei hepe-like virus 3; LHHEV, La Herrería hepevirus; MFHEV, Murine feces-associated hepe-like virus; NGSHEV, Nanhai ghost shark hepevirus; RaHEV, Rana hepevirus; RHEV, rat hepatitis E virus; SFHEV, Sogatella furcifera hepe-like virus; ShuAV, Shuangao alphatetra-like virus 1; WHEV, Wenzhou hepe-like virus 1; WTSHEV, Wenling thamnaconus striatus hepevirus.

## Supplementary Materials

The following are available online at https://www.mdpi.com/2076-2607/8/12/1869/s1.

## Appendix A. Description of the Novel Viruses of Interest Found in Nouragues and La Herrería

A partially complete fragment showing similarity to Duck hepatitis A virus (DHAV) was sequenced in the metagenomic analysis of cloacal samples from Nouragues. A Rufous-throated Antbird (*Gymnopithys rufigula*) was identified by RT-PCR as the carrier of the virus. After a second deep sequencing analysis of this individual’s cloacal sample, we obtained a complete genome of 7645 b (provisionally named French Guiana picornavirus; FGPV). The highest identity values in the amino acid sequence of the putative capsid protein (P1) of FGPV were 32.23% with *Avihepatovirus* and 31.04% with *Avisivirus*. However, the identity values for the 2C + 3CD amino acid sequence were higher, reaching 47.75% with *Avihepatovirus* and 38.30% with *Avisivirus*. A phylogenetic tree based on the amino acid sequence of the P1 region placed FGPV in a divergent branch within *Picornaviridae* family, sister to the clade grouping the genera *Avihepatovirus*, *Avisivirus*, *Aalivirus* and *Orivirus*, all of them detected only in avian species (Figure A1). Taking all these results together, the novel FGPV could not be unambiguously considered as part of the existing *Avihepatovirus* or *Avisivirus* genera according to ICTV criteria as identity for P1 is < 33%, but 2C + 3D is > 36%.

In the deep sequencing analysis from both regions, we identified three complete or almost complete viral genomes that, although they showed some similarity to families of animal virus, they were highly divergent from their closest relatives: a reo-like virus in Nouragues and two hepe-like viruses, one at each location.

As for the reo-like virus, we first obtained a partially complete genome showing similarity to this group of viruses. A primer set was designed to identify the carrier of the virus, a Wedge-billed Woodcreeper (*Glyphorynchus spirurus*) whose individual cloacal sample was subjected to a second deep sequencing analysis to obtain the complete genome of the novel reovirus. We finally obtained six complete fragments, for a total of 18,567 bp (approximately 75% of the complete genome of other reoviruses). These complete fragments showed similarity to Cimodo virus (Genbank accession number KF880748), an unclassified reovirus. The phylogenetic tree using the amino acid sequences of the putative RNA-dependent RNA polymerase (RdRp) showed that this novel reovirus, provisionally named French Guiana Reovirus (FGRV), is grouped with Cimodo virus in a divergent clade within the subfamily *Spinareovirinae* (Figure A2).

On the other hand, a complete and a partial genome, both showing similarity to hepevirus, were sequenced in the metagenomic analyses from Nouragues and La Herrería respectively. We carried out RT-PCRs using specific primers for each of these viruses to identify the positive individuals: a Plain Xenops (*Xenops minutus*) for French Guiana Hepevirus (FGHEV); and a European Robin (*Erithacus rubecula*) for La Herrería Hepevirus (LHHEV). The Rufous-throated Antbird was also positive for astrovirus and FGPV. The complete genome of FGHEV was 7595 b, unusually longer than other members of the family *Hepeviridae* [69]. Also unusually long is ORF1 (6455 b), encoding the putative non-structural polyprotein, which shows the conserved methyl-transferase, helicase and RdRp domains but has an extensive coding region of unknown function between RdRp and the stop codon (Figure A3). The analysis using Phyre2 showed that this region located at the end of ORF1 has 43% similarity (48.7% confidence) to PTPA-like superfamily, which groups protein tyrosine phosphatases. The arrangement of ORF2 in FGHEV, completely embedded in ORF1, is different from the layout of these two ORFs in hepeviruses, where they are completely separated (Figure A4). These characteristics, however, are common in the recently described hepe-like virus group, a sister clade to *Hepeviridae*. LHHEV, though incomplete, share some of these features with FGHEV. ORF1 is unusually long and ORF2 appears to overlap with it, in a different reading frame (Figure A4). Moreover, the incomplete LHHEV genome is 5451 b-long, which would suggest that the complete genome is also unusually long for a hepevirus. Similarity analyses carried out with RAT software along the ORF1 sequences for both viruses show that similarity with other hepe and hepe-like viruses, although low in general, peaks at the conserved motifs (Figure A5 and Figure A6).

Phylogenetic analyses place LHHEV as a sister taxon to murine feces associated and Hubei hepe-like viruses, either using RdRp amino acid sequence or the whole ORF1 (Figure A7). On the other hand, the phylogenetic location of FGHEV is less clear. When using RdRp, it is grouped in a basal group with Elicom virus and Barns Ness breadcrumb sponge hepe-like virus 1. However, when the whole ORF1 amino acid sequence is selected to infer the phylogeny, FGHEV is located halfway between hepe-like viruses and actual hepeviruses (Figure A7).
