# Supplementary material for: Comparative Metagenomics of Palearctic and Neotropical Avian Cloacal Viromes Reveal Geographic Bias in Virus Discovery
Source: Microorganisms. 2020 Nov 26;8(12):1869. doi: 10.3390/microorganisms8121869 (PMC7761369; doi:10.3390/microorganisms8121869)
Supplement: Supplementary file 1 [file microorganisms-08-01869-s001.pdf]

Supplementary material

Table S1. List of avian species including the 50 individuals selected for deep sequencing from Nouragues Natural Reserve.

Species	Order	Number of individuals
Glyphorynchus spirurus	Passeriformes	8
Gymnopithys rufigula	Passeriformes	2
Hylophylax naevius	Passeriformes	2
Lepidotrix serena	Passeriformes	2
Leptotila rufaxilla	Columbiformes	1
Mionectes macconnelli	Passeriformes	2
Philydor erythrocercum	Passeriformes	3
Bucco capensis	Piciformes	1
Chloroceryle inda	Coraciiformes	1
Corapipo gutturalis	Passeriformes	1
Corythopis torquatus	Passeriformes	1
Dixiphia pipra	Passeriformes	2
Myrmotherula longipennis	Passeriformes	1
Pithys albifrons	Passeriformes	2
Ramphocelus carbo	Passeriformes	2
Cyanocompsa cyanoides	Passeriformes	1
Formicarius analis	Passeriformes	1
Willisornis poecilinotus	Passeriformes	1
Micrastur ruficollis ssp. *concentricus*	Falconiformes	1
Myrmoderus ferrugineus	Passeriformes	2
Isleria guttata	Passeriformes	1
Ceratopipra erithrocephala	Passeriformes	1
Platyrinchus coronatus	Passeriformes	1
Platyrinchus saturatus	Passeriformes	1
Thamnomanes ardesiacus	Passeriformes	2
Tachyphonus surinamus	Passeriformes	1
Terenotriccus erythrurus	Passeriformes	1
Thamnomanes caesius	Passeriformes	1
Epinecrophylla gutturalis	Passeriformes	1
Turdus albicollis	Passeriformes	1
Xenops minutus	Passeriformes	1
Xiphorhynchus pardalotus	Passeriformes	1

Table S2. List of avian species including the 50 individuals selected for deep sequencing from La Herrería forest.

**Species**	**Order**	**Number of individuals**
Aegithalos caudatus	Passeriformes	1
Coccothraustes coccothraustes	Passeriformes	2
Dendrocopos minor	Piciformes	2
Dendrocopos major	Piciformes	1
Erithacus rubecula	Passeriformes	4
Ficedula hypoleuca	Passeriformes	4
Fringilla coelebs	Passeriformes	4
Luscinia megarhynchos	Passeriformes	1
Garrulus glandarius	Passeriformes	1
Cyanistes caeruleus	Passeriformes	4
Parus major	Passeriformes	2
Troglodytes troglodytes	Passeriformes	1
Sturnus unicolor	Passeriformes	1
Phylloscopus bonelli	Passeriformes	1
Sitta europaea	Passeriformes	1
Sylvia atricapilla	Passeriformes	10
Turdus merula	Passeriformes	8
Turdus philomelos	Passeriformes	2

Table S3. Primer sequences and the annealing temperature (Tm) for each specific primer pair utilized for the screening of the novel viruses. All RT-PCRs were carried out using the Verso 1-step RT-PCR kit (Thermo Fisher Scientific, USA) in a final volume of 25 µl per tube following manufacturer's instructions. The PCR protocol included an initial step at 50 ºC for 15 min for cDNA synthesis followed by 95 ºC during 15 min to inactivate the retrotranscriptase. Then, 35 cycles of denaturation at 95 ºC for 20 s, annealing at the specific Tm for 30 s and extension at 72 ºC for 1 min. The final extension took place at 72 ºC for 5 min. FGHEV, French Guiana hepevirus; FGPV, French Guiana picornavirus; FGRV, French Guiana reovirus; LHHEV, La Herrería hepevirus

Virus	Primer sequences	Tm
FGHEV	*Hepevirus196F:*<br>*5´-GTTCCAGAGAAGGGTTTGAG-3´*<br>*Hepevirus196R:*<br>*5´-CTCGTTGGGAACTTTTGCAG-3´*	58 ºC
FGPV	*Picornavirus434F:*<br>5´-GGCCCGCCTAATGCACAAGAAC-3´<br>*Picornavirus434R:*<br>5´-GGCCCGCCTAATGCACAAGAAC-3´	55 ºC
FGRV	*Reovirus2034F*:<br>5´-ACTGCCGACTGCTGTAGGAATGT-3´<br>*Reovirus2034R*:<br>5´-TTTGCGGCAGCGAAGAATAAT-3´	52 ºC
LHHEV	*HepeHerreriaF:*<br>5´-CAGGGTATATCTGCTTGGTC-3´<br>*HepeHerreriaR:*<br>5´-AGTTTCCACTTCTTACGCAT-3´	53 ºC
